# Surgical Treatment for Rectocele by Posterior Colporrhaphy Compared to Stapled Transanal Rectal Resection

**DOI:** 10.3390/jcm12020678

**Published:** 2023-01-15

**Authors:** Ohad Gluck, Doraid Matani, Ada Rosen, Elad Barber, Eran Weiner, Shimon Ginath

**Affiliations:** 1 Department of Obstetrics and Gynecology, Wolfson Medical Center, Holon 58100, Israel; 2 Sackler Faculty of Medicine, Tel Aviv University, Tel Aviv 69978, Israel; 3 Proctology and Pelvic Floor Unit, Division of General Surgery, Wolfson Medical Center, Holon 58100, Israel

**Keywords:** rectocele, obstructed defecation syndrome, colporrhaphy

## Abstract

Background: Rectocele is defined as a defect in the rectovaginal septum, causing symptoms like obstructed defecation syndrome (ODS), vaginal bulging, etc. Once the rectocele is larger than 3 cm and/or symptomatic, surgery should be considered. The surgical approach can be either transvaginal, transanal or transperineal. Two of the most common procedures in treating rectocele are posterior colporrhaphy (PC) and stapled trans anal rectal resection (STARR). The purpose of this study was to compare surgical outcomes of both procedures. Methods: This is a retrospective cohort study. Included were patients of the age of 18–85 years that underwent either STARR (n = 49 patients) or PC (n = 24 patients) procedures after a full clinical (defecography and physical exam before and after the surgery) and physiologic (a detailed questionnaire before and after the surgery) surveys. Symptoms of ODS before and after surgery were evaluated by questioners. Results: Preoperatively, the patients in the STARR group had significantly higher rates of ODS: straining (90.9% vs. 65.2%), incomplete evacuation (100% vs. 69.6%), hard stool (57.8% vs. 43.5%), sense of obstruction (76.1% vs. 56.5%), and use of digitation (64.4% vs. 47.8%), or laxatives (70% vs. 47.8%), *p* < 0.001. Anatomically, the mean rectocele size was smaller for the STARR group, compared to the PC group (3.8 ± 1.4 vs. 5.3 ± 2.2 cm, respectively, *p* < 0.001). Postoperatively, in the STARR group, higher rates of patients complained about straining (36.4% vs. 21.7%, *p* < 0.001) and use of digitation (64.4% vs. 26.1%, *p* < 0.001), whereas lower rates of patients complained about incomplete evacuation (41.2% vs. 56.5%, *p* = 0.05) and sense of obstruction (17.6%, vs. 34.8%, *p* = 0.03), compared to the PC group. Among patients who underwent the STARR procedure, a decrease in rates of all symptoms was noted (straining 54.5%, incomplete evacuation 58.8%, hard stool 29.2%, sense of obstruction 58.5%, use of digitation 0.1%, and use of laxatives 31.5%). Both procedures are effective in reducing rectocele size (STARR- 1.9 ± 1 cm, PC- 3.1 ± 1). Conclusions: Both STARR and PC are effective in treating rectocele. It seems that the STARR procedure is superior to the PC procedure in treating symptoms of ODS.

## 1. Introduction

Rectocele is a bulging of the rectum into the vaginal cavity caused due to posterior compartment damage with weakness of posterior vaginal wall support [1]. Although up to 93% of healthy, asymptomatic women are found on defecating proctography to have radiological evidence of a rectocele, the estimated incidence of symptomatic rectocele is four per 1000 women, and it is most common in the elderly or multi-parous [2]. True rectoceles are found in more than half of women presenting with pelvic floor disorders [2]. Moally et al. [3] reported that younger age at first delivery, higher body mass index (BMI), forceps delivery, and previous gynecologic surgery were significantly associated with subsequent development of pelvic floor disorders. Rectocele might adversely affect the quality of life, with symptoms of vaginal bulging and/or obstructive defecation syndrome (ODS) [1,4]. 

Symptoms of ODS include rectal or lower abdominal pain, incomplete rectal evacuation, the use of digitation or perineal manipulation to help the defecation, straining, spending prolonged time in toilet, perineal descent, report of hard stools, as well as dependency on laxatives and enemas [5].

Surgery is usually indicated when conservative treatments have failed, with the aim of restoring normal anatomy and function [1,6].

Surgical treatment can be abdominal, transperineal, transvaginal, or transanal [4] (Table 1). The abdominal techniques include rectopexy and colorectum resection, the transperineal techniques are the Alteimer’s rectosigmoidectomy and the Delorme’s procedure, the main transvaginal technique is posterior colporrhaphy (PC), and the transanal procedures include the Sullivan–Khubchandani technique, the stapled transanal rectal resection (STARR) [1,4]. The STARR procedure, which was introduced by Longo in 2004, consists of an endorectal resection of the distal rectum using a stapler [7].

Both transvaginal and transrectal surgical techniques are effective when treating posterior compartment defects [1]. However, additional data are needed in order to tailor the treatment to each patient, personally.

Therefore, we aimed to compare surgical outcomes of rectocele repair, between STARR and PC.

## 2. Materials and Methods

This was a retrospective study. The study was approved by the local review board (WOMC-0062-09). Included were all patients who underwent eighter STARR or PC at our institute, in the indication of symptomatic rectocele, between January 2010–December 2019. Exclusion criteria were patients with additional anal or vaginal pathology and patients who underwent additional concurrent procedures (additional procedures included surgical corrections of the anterior and/or apical vaginal compartment prolapse, and anti-urinary incontinence procedures).

Prior to surgery, the patients were examined and asked to complete a questionnaire (Appendix A). Then, a multidisciplinary team, including a urogynecologist and a general surgeon, came to a shared decision regarding the appropriate intervention for each patient. The STARR was performed by a single general surgeon, whereas the PC was performed by a single urogynecologist.

Patients were also examined and completed a questionnaire at a follow-up visit, 8 weeks after the surgery. Anterior rectocele size was measured during PR examination (performed by the surgeon) before surgery and afterward at the follow-up visit [6].

The study population was divided according to the surgery performed: STARR and PC groups. Treatment success was defined as the resolution of symptoms (vaginal bulging and/or obstructive defecation syndrome). We compared the success rate, background characteristics, and operative details between the groups.

### Statistical Analysis

The primary outcome was defined as the success rate of surgical treatment evaluated by the presence of straining effort and incomplete evacuation of stool. Data were analyzed using SPSS statistical analysis software v23.0 (IBM Inc., Armonk, NY, USA). Continuous variables were compared by the ANOVA test, and categorical variables were compared by chi-square test. All tests were two sided, and a *p*-value < 0.05 was considered statistically significant.

## 3. Results 

Overall, 49 patients (67.1%) were included in the STARR group and 24 patients (32.9%) in the PC group. The patients in the STARR group were younger, compared to the PC group (54.2 ± 9.7 vs. 59.3 ± 10.7 years, respectively, *p* = 0.04) and had experienced ODS for a longer period of time (36.8 ± 34.1 vs. 17.6 ± 8.2, months, respectively, *p* = 0.008), compared to the PC group. Preoperatively, the patients in the STARR group had significantly higher rates of ODS: straining (90.9% vs. 65.2%), incomplete evacuation (100% vs. 69.6%), hard stool (57.8% vs. 43.5%), sense of obstruction (76.1% vs. 56.5%), and use of digitation (64.4% vs. 47.8%) or laxatives (70% vs. 47.8%), *p* < 0.001.

Anatomically, the mean rectocele size was smaller for the STARR group, compared to the PC group (3.8 ± 1.4 vs. 5.3 ± 2.2 cm. respectively, *p* < 0.001) (Table 2).

Postoperatively, in the STARR group, higher rates of patients complained about straining (36.4% vs. 21.7%, *p* < 0.001) and use of digitation (64.4% vs. 26.1%, *p* < 0.001), whereas lower rates of them complained about incomplete evacuation (41.2% vs. 56.5%, *p* = 0.05) and sense of obstruction (17.6%, vs. 34.8%, *p* = 0.03), compared to the PC group (Table 3). Anatomical outcomes and overall satisfaction were comparable between the groups (Table 3).

Among patients who underwent the STARR procedure, a decrease in rates of all symptoms was noted (straining 54.5%, incomplete evacuation 58.8%, hard stool 29.2%, sense of obstruction 58.5%, use of digitation 58.5%, and use of laxatives 31.5%) (Table 4). On the other hand, among the PC group, a decrease was observed only in symptoms of straining (43.5%) and use of digitation (21.7%), as well as in evacuation time (61.8 ± 20 min) (Table 5). Both procedures are effective in reducing rectocele size (STARR-1.9 ± 1 cm, PC-3.1 ± 1) (Table 4 and Table 5).

We also calculated the rates of composite outcome, which was defined as any of the three complaints: straining, incomplete evacuation, or use of digitation. There was no difference between STARR and PC groups in the rate of composite outcome before and after the surgery (Table 2 and Table 3).

The rate of composite outcome preoperatively was significantly higher, comparing to the rate of composite outcome postoperatively, for both the STARR group (100% vs. 56.3%, *p* < 0.001) and the PC group (91.3% vs. 60.9%, *p* = 0.03) (Table 4 and Table 5).

## 4. Discussion

In the current study, we compared two surgical techniques for treating rectocele: STARR and PC. Preoperatively, we found that although the rate of ODS symptoms was higher among patients who underwent STARR, the mean rectocele size was smaller, compared to PC.

This difference could imply the leading indication for each of the procedures: whereas patients who suffer from ODS symptoms are more often been offered to undergo STARR, those who suffer from vaginal bulging are more likely to undergo PC.

As for the surgical outcomes, we observed that both procedures were effective in reducing rectocele size and overall satisfaction, whereas the STARR procedure was more effective in reducing the rates of ODS symptoms.

This could be explained by the surgical technique: in the STARR procedure, a circular resection of redundant rectal wall tissue is performed, in a manner that restores the anatomical rectal lumen. However, in the PC, only the rectovaginal facia is being corrected, so cases of rectal intussusception might be left untreated [1].

As in other studies [8,9,10], we also demonstrated that STARR surgical procedure has been proven to be safe and effective in treating symptoms of ODS. However, the literature regarding the best procedure to treat rectocele is inconclusive. Although trans-perineal procedures (such as STARR) were demonstrated to be effective, there is a distinct lack of data comparing transvaginal procedures (such as PC) to other approaches [1]. The current study has a few strengths: First, to the best of our knowledge, this is the first study that compares satisfaction and objective outcomes between two of the most common procedures for treating rectocele: STARR and PC. Second, we used a uniform questionnaire to evaluate objective outcomes among the two study populations.

Our study is not free of limitations: First, as this is a retrospective study, it is potentially subjected to a selection bias. It is possible that patients with ODS symptoms were referred in more cases to proctologists, and therefore were offered to undergo STARR, which is usually performed by general surgeons (proctologists), rather than urogynecologists. On the other hand, patients who suffered from vaginal bulging were more likely to seek treatment in urogynecology clinics, where they were offered to undergo PC, rather than STARR. Therefore, despite the vivid superiority of the STARR over the PC, in reducing the rates of ODS symptoms, the satisfaction rate was similar between the groups. Second, in this study, we were unable to assess long-term outcomes, including recurrence rates. Lastly, our sample size is relatively small. It is possible that in a larger population we might have been able to demonstrate differences also in anatomical outcomes.

## 5. Conclusions

We showed that both STARR and PC procedures are effective in treating rectocele, objectively and subjectively. In addition, it seems that STARR is superior to PC, in improving ODS symptoms among patients with rectocele. Further studies are needed to prospectively compare the long-term efficacy and recurrence rate of these two procedures.

## Figures and Tables

**Table 1:** Surgical procedures to correct rectocele [4,8].

	Year of Introduction	Approach	Description	Success Rate
Rectopexy	1952	Abdominal	Fixing the pararectal tissue to the promontory by mesh	90–95%
Alteimer’s procedure	1970	Perineal	perineal rectosigmoidectomy	80%
Delorme’s procedure	1900	Perineal	Rectal mucosa resection	70–75%
posterior colporrhaphy	1900	Vaginal	Plication of the recto-vaginal fascia	76–96%
Sullivan–Khubchandani	1983	Anal	Rectal plication using staples and sutures	63–82%
Stapled transanal rectal resection	1997	Anal	endorectal resection of the distal rectum	70–96%

**Table 2:** Preoperative evaluation of the study groups.

	STARR (n = 49)	PC (n = 24)	*p*
Mean age *	54.2 ± 9.7	59.3 ± 10.7	0.04
Duration (Months) *	36.8 ± 34.1	17.6 ± 8.2	0.008
Straining	40/44 (90.9%)	15/23 (65.2%)	*p* < 0.001
Incomplete evacuation	46/46 (100%)	16/23 (69.6%)	*p* < 0.001
Hard Stool	26/45 (57.8%)	10/23 (43.5%)	*p* < 0.001
Sense of obstruction	35/46 (76.1%)	13/23 (56.5%)	*p* < 0.001
Use of digitation	29/45 (64.4%)	11/23 (47.8%)	*p* < 0.001
Use of laxatives	28/40 (70%)	11/23 (47.8%)	*p* < 0.001
Perineal descent (cm) * Evacuation time (s) *	1.4 ± 1.4 73.5 ± 42.2	1.7 ± 0.9 99.6 ± 41.4	0.6 0.07
Rectocele size (cm) *	3.8 ± 1.4	5.3 ± 2.2	*p* < 0.001
Composite outcome	44/44 (100%)	21/23 (91.3)	0.1

* Mean ± SD; composite outcome: any of the following complaints: straining, incomplete evacuation, or use of digitation.

**Table 3:** Postoperative evaluation of the study groups.

	STARR (n = 49)	PC (n = 24)	*p*
Straining	12/32 (36.4%)	523/(21.7%)	*p* < 0.001
Incomplete evacuation	14/34 (41.2%)	13/23 (56.5%)	0.05
Hard Stool	8/28(28.6%)	7/23 (30.4%)	0.31
Sense of obstruction	6/34 (17.6%)	8/23 (34.8%)	0.03
Use of digitation	2/34(64.4%)	6/23 (26.1%)	*p* < 0.001
Use of laxatives	10/26 (38.5%)	7/23 (30.4%)	0.26
Perineal descent (cm) *	0.8 ± 1.2	1.1 ± 0.6	0.69
Evacuation time (s) *	49.6 ± 36.9	37.8 ± 21.5	0.48
Rectocele size (cm) *	1.9 ± 1	2.2 ± 1	0.51
Overall satisfaction	79% ± 40	80% ± 40	0.67
Composite outcome	18/32 (56.3%)	14/23 (60.9%)	0.78

* Mean ± SD; composite outcome: any of the following complaints: straining, incomplete evacuation, or use of digitation.

**Table 4:** Presurgical vs. postsurgical evaluation in the STARR group.

	STARR/Pre-Surgery	STARR/Post-Surgery	Improvement	*p*
Straining	40/44 (90.9%)	12/32 (36.4%)	54.50%	*p* < 0.001
Incomplete evacuation	46/46 (100%)	14/34 (41.2%)	58.80%	*p* < 0.001
Hard Stool	26/45 (57.8%)	8/28 (28.6%)	29.20%	*p* < 0.001
Sense of obstruction	35/46 (76.1%)	6/34 (17.6%)	58.50%	*p* < 0.001
Use of digitation	29/45 (64.4%)	2/34 (5.9%)	58.50%	*p* < 0.001
Use of laxatives	28/40 (70%)	10/26 (38.5%)	31.50%	*p* < 0.001
Perineal descent (cm) *	1.4 ± 1.4	0.8 ± 1.2	0.6	0.2
Evacuation time (s) *	73.5 ± 42.2	49.6 ± 36.9	23.9	0.1
Rectocele size (cm) *	3.8 ± 2	1.9 ± 1	1.9 ± 1	*p* < 0.001
Composite outcome	44/44 (100%)	18/32 (56.3%)	43.70%	*p* < 0.001

* Mean ± SD; composite outcome: any of the following complaints: straining, incomplete evacuation, or use of digitation.

**Table 5:** Preoperative vs. postoperative evaluation in the PC group.

	PC Pre-Surgery	PC Post-Surgery	Improvement	*p*
Straining	15/23 (65.2%)	523/(21.7%)	43.50%	*p* < 0.01
Incomplete evacuation	16/23 (69.6%)	13/23 (56.5%)	3/23 (13.1%)	0.4
Hard Stool	10/23 (43.5%)	7/23 (30.4%)	3/23 (13.1%)	0.2
Sense of obstruction	13/23 (56.5%)	8/23 (34.8 %)	5/23 (21.7%)	0.07
Use of digitation	11/23 (47.8%)	6/23 (26.1 %)	21.70%	0.04
Use of laxatives	11/23 (47.8%)	7/23 (30.4%)	4/23 (17.4%)	0.13
Perineal descent (cm) *	1.7 ± 0.9	1.1 ± 0.6	0.6	0.3
Evacuation time (s) *	99.6 ± 41	37.8 ± 21	61.8 ± 20	0.03
Rectocele size (cm) *	5.3 ± 2	2.2 ± 1	3.1 ± 1	0.04
Composite outcome	21/23 (91.3%)	14/23 (60.9%)	30.40%	0.03

* Mean ± SD; composite outcome: any of the following complaints: straining, incomplete evacuation, or use of digitation.

## Data Availability

data is unavailable due to privacy.

## Appendix A

**Table A1:** Questionnaire used to evaluate obstructed defecation syndrome symptoms.

Straining	Yes/No
Incomplete evacuation	Yes/No
Hard Stool	Yes/No
Sense of obstruction	Yes/No
Use of digitation	Yes/No
Use of laxatives	Yes/No
Evacuation time (s)	
Overall satisfied for having the surgery *	Yes/No

* Post-surgical only.
